# HTLV-1 bZIP factor enhances TGF-beta signaling through p300 coactivator

**DOI:** 10.1186/1742-4690-8-S1-A142

**Published:** 2011-06-06

**Authors:** Tiejun Zhao, Yorifumi Satou, Kenji Sugata, Patrick L Green, Takeshi Imamura, Masao Matsuoka

**Affiliations:** 1 Laboratory of Virus Control, Institute for Virus Research, Kyoto University, Kyoto, Japan; 2 Center for Retrovirus Research and Departments of Veterinary Biosciences and Molecular Virology, Immunology and Medical Genetics, The Ohio State University, Columbus, OH, USA; 3 Department of Molecular Medicine for Pathogenesis, Ehime University Graduate School of Medicine, Shitsukawa, Toon, Ehime, Japan; 4 Division of Biochemistry, the Cancer Institute of the Japanese Foundation for Cancer Research (JFCR), Tokyo, Japan; 5 Core Research for Evolutional Science and Technology (CREST), Japan Science and Technology Agency (JST), Kawaguchi-shi, Saitama, 332-0012, Japan

## 

Adult T-cell leukemia (ATL) is a neoplastic disease caused by Human T-cell leukemia virus type 1 (HTLV-1). ATL cells possess a CD4+CD25+ phenotype, similar to that of regulatory T cells (Treg). The HTLV-1 bZIP factor (HBZ), which is consistently expressed in ATL, has a critical role in the development of ATL and HAM/TSP. In the present study, we found that HBZ enhanced TGF-beta/Smad transcriptional activity in a manner dependent on p300. Co-immunoprecipitation assay confirmed that HBZ interacted with Smad3, and formed a ternary complex with Smad3 and p300. In the presence of HBZ, the interaction between Smad3 and p300 was enhanced. The N-terminal LXXLL motif of HBZ was essential for HBZ-mediated TGF-beta signaling activation, while Smad3 interacted with HBZ through its C-terminal MH2 domain. Furthermore, physiological level of HBZ could rescue the repressed TGF-beta responses by Tax. We also found that HBZ activated transcription of the Foxp3 gene through its Smad site. Our study shows that HBZ enhances TGF-beta signaling while Tax suppresses this pathway, and this enhancing activity leads to transcription of Foxp3 gene.

